# Interval cancers in the Dutch breast cancer screening programme

**DOI:** 10.1038/sj.bjc.6690786

**Published:** 1999-11

**Authors:** J Fracheboud, H J de Koning, P M M Beemsterboer, R Boer, A L M Verbeek, J H C L Hendriks, B M van Ineveld, M J M Broeders, A E de Bruyn, P J van der Maas

**Affiliations:** National Evaluation Team for Breast cancer screening (NETB) Departments of Public Health andHealth Policy and Management, Erasmus University Rotterdam, PO Box 1738, Rotterdam DR 3000, The Netherlands; Departments of Epidemiology, Radiology, University of Nijmegen, P.O. Box 9101, Nijmegen HB 6500, The Netherlands

**Keywords:** breast cancer, interval cancer, proportionate incidence, population-based screening, evaluation

## Abstract

The nationwide breast cancer screening programme in The Netherlands for women aged 50–69 started in 1989. In our study we assessed the occurrence and stage distribution of interval cancers in women screened during 1990–1993. Records of 0.84 million screened women were linked to the regional cancer registries yielding a follow-up of at least 2.5 years. Age-adjusted incidence rates and relative (proportionate) incidences per tumour size including ductal carcinoma in-situ were calculated for screen-detected and interval cancers, and cancers in not (yet) screened women, comparing them with published data from the UK regions North West and East Anglia. In total 1527 interval cancers were identified: 0.95 and 0.99 per 1000 woman-years of follow-up in the 2-year interval after initial and subsequent screens respectively. In the first year after initial screening interval cancers amounted to 27% (26% after subsequent screens) of underlying incidence, and in the second year to 52% (55%). Generally, interval cancers had a more favourable tumour size distribution than breast cancer in not (yet) screened women. The Dutch programme detected relatively less (favourable) invasive cancers in initial screens than the UK programme, whereas the number of interval cancers confirms UK findings. Measures should be considered to improve the detection of small invasive cancers and to reduce false-negative rates, even if this will lead to increasing referral rates. © 1999 Cancer Research Campaign


*British Journal of Cancer* (1999) **81**(5), 912–917
© 1999 Cancer Research Campaign
Article no. bjoc.1999.0786

### Interval cancers in the Dutch breast cancer screening programme

**J Fracheboud[1], HJ de Koning[1], PMM Beemsterboer[1], R Boer[1], ALM Verbeek[3], JHCL Hendriks[4], BM van Ineveld[2], MJM Broeders[3], AE de Bruyn[1] and PJ van der Maas[1]**

National Evaluation Team for Breast cancer screening (NETB) Departments of [1]Public Health and [2]Health Policy and Management, Erasmus University Rotterdam, PO Box 1738, 3000 DR Rotterdam, The Netherlands; Departments of [3]Epidemiology and [4]Radiology, University of Nijmegen, P.O. Box 9101, 6500 HB Nijmegen, The Netherlands

**Summary** The nationwide breast cancer screening programme in The Netherlands for women aged 50–69 started in 1989. In our study we assessed the occurrence and stage distribution of interval cancers in women screened during 1990–1993. Records of 0.84 million screened women were linked to the regional cancer registries yielding a follow-up of at least 2.5 years. Age-adjusted incidence rates and relative (proportionate) incidences per tumour size including ductal carcinoma in-situ were calculated for screen-detected and interval cancers, and cancers in not (yet) screened women, comparing them with published data from the UK regions North West and East Anglia. In total 1527 interval cancers were identified: 0.95 and 0.99 per 1000 woman-years of follow-up in the 2-year interval after initial and subsequent screens respectively. In the first year after initial screening interval cancers amounted to 27% (26% after subsequent screens) of underlying incidence, and in the second year to 52% (55%). Generally, interval cancers had a more favourable tumour size distribution than breast cancer in not (yet) screened women. The Dutch programme detected relatively less (favourable) invasive cancers in initial screens than the UK programme, whereas the number of interval cancers confirms UK findings. Measures should be considered to improve the detection of small invasive cancers and to reduce false-negative rates, even if this will lead to increasing referral rates. © 1999 Cancer Research Campaign

**Keywords:** breast cancer; interval cancer; proportionate incidence; population-based screening; evaluation

Interval cancers are an important indicator of the quality of a breast cancer screening programme and a predictor for its success in reducing breast cancer mortality (Day et al, 1995). The occurrence of interval cancers in screening trials and experimental programmes has been well documented (Tabár et al, 1987; Peeters et al, 1989; Brekelmans et al, 1992; Moss et al, 1993; Vitak et al, 1997). As routine screening programmes have only started relatively recently, there is still limited information on interval cancers in such programmes (Woodman et al, 1995; Faux et al, 1997; Klemi et al, 1997; Sylvester et al, 1997; Boer et al, 1998; Schouten et al, 1998). First published regional interval cancer rates from the UK programme were higher than expected and led to some commotion about the performance of the screening programme (Woodman et al, 1995).

During 1989–1997 a nationwide breast cancer screening programme was established in The Netherlands. At the time it was estimated that it would reduce breast cancer mortality in the total female population by 17% (Koning et al, 1995b). Early findings up to 1996 with regard to participation, detection of breast cancers and stage distribution of screen-detected cancers were rather favourable, especially in initial screens (Koning et al, 1995*a*; Fracheboud et al, 1998). However, the interpretation of the results was hampered by the lack of information on interval cancers. In this paper, we present the first national figures on the occurrence of interval cancers and compare these to regional data from the UK.

*Received 25 January 1999*
*Revised 21 April 1999*
*Accepted 29 April 1999*

*Correspondence to:* J Fracheboud

### MATERIALS AND METHODS

### The Dutch national breast cancer screening programme

The nationwide breast cancer screening programme started in the period 1990–1991 in nine screening regions, and at the end of 1993 covered 69% of the target population in The Netherlands. Details of the programme, offering a biennial screening mammography for women aged 50–69 years, are described elsewhere (Koning et al, 1995*a*; Fracheboud et al, 1998). In the period 1990–1993, 1.1 million women aged 50–69 had been invited for screening and 0.84 million women had a screening examination (attendance rate 76%).

The nine regional screening organizations provide annually a data set to the National Evaluation Team for Breast cancer screening (NETB). The NETB aggregates the regional data to national files for further analysis and comparison with the expectations based on the cost-effectiveness analysis (Koning et al, 1991). For evaluation purposes screen examinations were subdivided into initial and subsequent screens. An initial screen is defined as the first time the woman is screened within the breast cancer screening programme; a subsequent screen as a rescreen performed within 2.5 years since the previous screen. Subsequent screens performed after a longer interval ($n$ = 5357) were excluded from analysis of screen-detected and interval cancers. Date of diagnosis of breast cancer is the day of (diagnostic) biopsy. Reference date of the age definition is 1 January of the year of screening.

### Identifying interval cancers

Interval cancers were identified by linking regional records of women screened during 1990–1993 to the regional cancer registries, comparing birth date, first four characters of surname and postal code in both files. Positive matches were manually checked to exclude screen-detected cancers of a later screening round. Due to an inevitable delay in the cancer registry and because of the screening interval of 2 years, records of women screened in a certain calendar year (e.g. 1993) cannot be linked to cancer registry records earlier than in the third year after screening (thus 1996 or later). Although the national cancer registry has reached a 100% coverage in The Netherlands since 1989 (NCR, 1992), for technical reasons the linkage procedure was carried out at regional level. This may lead to some underreporting of interval cancers in women diagnosed and treated in another region than where screening took place. The mean national under-reporting is estimated to be approximately 4%. Furthermore, a small proportion of screened women did not give authorization for record linkage (0.07% of all screenees).

Within the national evaluation system breast cancers are defined as epithelial invasive cancers or ductal carcinoma in situ (DCIS) of the breast. Lobular carcinomata in-situ are regarded as benign lesion and therefore excluded from analysis. Interval cancers are breast cancers diagnosed in women after a negative screen (defined as no recommendation for referral) or after a positive screen in which assessment did not lead to the diagnosis of cancer, and before an eventual succeeding screen examination. In case of a simultaneously diagnosed second breast cancer only the one with the worst prognosis, and in consecutively diagnosed cases only the first one is taken into account. Tumour size is classified in accordance with the UICC 1987 guidelines. Percentage distributions of breast cancer size are based on all breast cancers, including DCIS (with the exception of Figure 1), Tx tumours and not classified cancers.

### Analysis

Interval cancer data were available nationally for the period 1990–1992 but for 1993, not from one of the nine regions. In consequence, all data from 1993 from this region were excluded from the analysis. Follow-up time was calculated from date of last screen until date of diagnosis of interval cancer, date of next screen examination or date of eventual death or moving out of the region. In women with screen-detected cancer, follow-up time was defined as zero. The observed screen-detected and interval cancer rates were compared with expected results based on outcomes of the MISCAN micro simulation model, serving as reference values for the national evaluation. The model simulates life histories in the absence of screening and calculates how they change after introduction of a screening programme depending on the chosen policy (Oortmarssen et al, 1990; de Koning, 1995*a*).

The underlying breast cancer incidence and stage distribution were derived from the first nationally available cancer registry data from 1989. From these, the data of the central (Utrecht) and the eastern (Nijmegen) regions were subtracted because they were

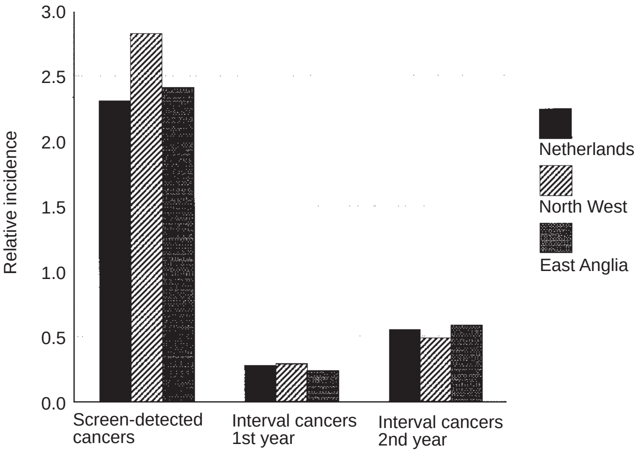

**Figure 1** Relative incidences of invasive screen-detected cancers and of invasive interval cancers (proportionate incidence) in the first and in the second year after initial screening, The Netherlands and the UK regions North West and East Anglia, women aged 50–64 years influenced by previous screening activities in the 1970s and 1980s. The resulting population figures served as reference population for age-adjusting of incidence rates by direct standardization. With respect to tumour size distribution of interval cancers, data were not separately available for interval cancers after initial or after subsequent screens. Breast cancer incidence and stage distribution in not (yet) screened women for the period 1990–1993 were estimated by subtracting the number of all screened women and the number of all screen-detected and interval cancers, including subsequent screens with a longer interval than 2.5 years, from the total population and incidence, respectively, in the corresponding period. The category of not (yet) screened women also includes women with breast cancer from whom it is not known whether they were screened or not (6% of all breast cancer cases). Age-adjusted incidence rates of screen-detected cancers, interval cancers and cancers in not (yet) screened women were related to the underlying incidence yielding relative incidences (or proportionate incidence with regard to interval cancers).

For the comparison with the North West and East Anglia regions in the UK screening programme, Dutch results were calculated for the age group 50–64 years in the same way as described above. Interval cancers were presented as proportionate incidences of the underlying incidence in the corresponding programme. Figures from the East Anglia and North West regions were derived from published data (Day et al, 1995; Woodman et al, 1995; Boer et al, 1998). In Figure 1 the analysis had to be restricted to invasive cancers only because data on DCIS was not available from the East Anglia programme.

### RESULTS

### Interval cancer incidence

In 553 501 initially screened women, 3635 breast cancers were detected, resulting in a detection rate of 6.57 per 1000 screened women (Table 1). In the first 2 years after screening 1002 interval cancers (invasive and in situ) were diagnosed, corresponding to an interval cancer incidence rate of 0.95 per 1000 woman-years of follow-up. For the 202 782 subsequent screens performed within

© 1999 Cancer Research Campaign

**Table 1** Breast cancer (invasive and in situ) incidence rates, either screen-detected or interval cancers by age per 1000 screened women in 1990–1993

| Age Years | Screened women | Screen-detected breast cancers | | Interval cancers (invasive and in situ) per 1000 woman-years follow-up by 6-months period after screening 0–23 | | < 6 | | 6–11 | | 12–17 | | 18–23 | | 24–29 | |
|---|---|---|---|---|---|---|---|---|---|---|---|---|---|---|---|
| | ***n*** | ***n*** | **/1000** | ***n*** | **/1000** | ***n*** | **/1000** | ***n*** | **/1000** | ***n*** | **/1000** | ***n*** | **/1000** | ***n*** | **/1000** |
| (After) initial screen | | | | | | | | | | | | | | | |
| 50–54 | 170 854 | 718 | 4.20 | 323 | 0.99 | 28 | 0.33 | 100 | 1.18 | 85 | 1.01 | 110 | 1.53 | 26 | 2.02 |
| 55–59 | 140 194 | 817 | 5.83 | 233 | 0.87 | 22 | 0.32 | 55 | 0.79 | 74 | 1.07 | 82 | 1.38 | 25 | 2.48 |
| 60–64 | 134 899 | 987 | 7.32 | 246 | 0.96 | 23 | 0.34 | 50 | 0.75 | 84 | 1.27 | 89 | 1.56 | 21 | 2.01 |
| 65–69 | 107 554 | 1113 | 10.35 | 200 | 0.96 | 23 | 0.43 | 42 | 0.79 | 61 | 1.16 | 74 | 1.53 | 45 | 1.58 |
| 50–69 | 553 501 | 3635 | 6.57 | 1002 | 0.95 | 96 | 0.35 | 247 | 0.90 | 304 | 1.12 | 355 | 1.50 | 117 | 1.89 |
| Expected | | | 6.50 | | 1.00 | | 0.50 | | 0.91 | | 1.22 | | 1.44 | | 1.52 |
| (After) Subsequent screen (performed within 2.5 years since previous screen) | | | | | | | | | | | | | | | |
| 50–54 | 49 711 | 120 | 2.41 | 81 | 0.87 | 10 | 0.41 | 18 | 0.73 | 38 | 1.58 | 15 | 0.75 | 8 | 1.96 |
| 55–59 | 54 101 | 191 | 3.53 | 105 | 1.04 | 13 | 0.48 | 20 | 0.75 | 43 | 1.65 | 29 | 1.34 | 8 | 1.88 |
| 60–64 | 57 477 | 199 | 3.46 | 103 | 0.95 | 7 | 0.25 | 18 | 0.63 | 41 | 1.47 | 37 | 1.59 | 3 | 0.60 |
| 65–69 | 41 493 | 191 | 4.60 | 88 | 1.11 | 12 | 0.58 | 23 | 1.12 | 25 | 1.24 | 28 | 1.55 | 12 | 1.20 |
| 50–69 | 202 782 | 701 | 3.46 | 377 | 0.99 | 42 | 0.42 | 79 | 0.79 | 147 | 1.50 | 109 | 1.31 | 31 | 1.33 |
| Expected | | | 4.30 | | 0.96 | | 0.45 | | 0.87 | | 1.19 | | 1.44 | | 1.68 |

[a]Based on the outcomes of linkage to the cancer registry in all nine regions 1990–1992 and in eight regions 1993.

**Table 2** Age-adjusted breast cancer (invasive and in situ) incidence rates per 1000 women, and relative (proportionate) incidences of underlying incidence by tumour size, women aged 50–69 years

| | (Screened) women | Breast cancers | | Relative (proportionate) incidence | | | | |
|---|---|---|---|---|---|---|---|---|
| | ***n*** | ***n*** | **per 1000** | **All** | **DCIS** | **T1a+b (≤ 10 mm)** | **T1c (11–20 mm)** | **T2+ (> 20 mm)** |
| Underlying incidence[a] | 1 208 643 | 2809 | 2.32 | 1 | 1 | 1 | 1 | 1 |
| Initial screens 1990–1993[b] | | | | | | | | |
| Detection rate | 553 501 | 3639 | 6.85 | 2.95 | 11.80 | 10.85 | 3.48 | 1.05 |
| Interval cancers 1st year[c] | 553 501 | 343 | 0.62 | 0.27 | 0.17 | 0.37 | 0.35 | 0.19 |
| Interval cancers 2nd year[c] | 553 501 | 659 | 1.21 | 0.52 | 0.33 | 0.81 | 0.54 | 0.44 |
| Subsequent screens 1990–1993[b] | | | | | | | | |
| Detection rate | 202 778 | 712 | 3.34 | 1.44 | 6.19 | 5.98 | 1.60 | 0.49 |
| Interval cancers 1st year[c] | 202 778 | 121 | 0.61 | 0.26 | 0.16 | 0.43 | 0.35 | 0.18 |
| Interval cancers 2nd year[c] | 202 778 | 256 | 1.27 | 0.55 | 0.34 | 0.88 | 0.57 | 0.45 |
| Breast cancers 1990–1993 not (yet) screened women[b] | 4 860 544 | 11 895 | 2.45 | 1.05 | 1.37 | 1.22 | 1.09 | 0.94 |

[a]Based on cancer registry data for 1989 from seven regions (two regions with pilot project excluded). [b]1990–1992: nine regions; 1993: eight regions.
[c]Data on tumour size distribution not separately available for interval cancers after initial and after subsequent screening.

2.5 years after the previous screen, these rates were 3.46 per 1000 screened women and 0.99 per 1000 woman-years respectively. This means that of all breast cancers diagnosed in regular participants 64% will be detected by screening, 36% will emerge as interval cancers. While detection rates show a clear age-dependency, interval cancer rates do not. Interval cancer rates increased with time after screening. In subsequent screens, the observed detection rate was distinctively lower and the interval cancer incidence rate higher than expected.

### Relative incidences

Table 2 presents age-adjusted breast cancer incidence rates and relative incidences of underlying incidence per tumour size, including DCIS. In 1989, the incidence in the not-screened Dutch population (underlying incidence) aged 50–69 was 2.32 per 1000. Initial screening during 1990–1993 led to the detection of almost 3 times, and subsequent screening to almost 1.5 times, as many cancers. In the first year after initial screening interval cancers were found in 27% (26% in subsequent screens) of the underlying incidence, and in the second year in 52% (55%). In 1990–1993, almost 12 000 breast cancers were diagnosed in not-screened women, resulting in an overall incidence rate of 2.45 per 1000, which is 5% higher than in the not-screened population in 1989. In situ and small invasive cancers were relatively more often diagnosed in this group than in 1989, while large invasive cancers were reported less frequently.

### Tumour size distribution

Table 3 gives the tumour size distribution by different incidence groups, based on the same numbers of breast cancers as in Table 2.

**Table 3** Age-adjusted tumour size distribution (%), women aged 50–69 years

| | Breast cancers | Per cent tumour size distribution | | | | | |
|---|---|---|---|---|---|---|---|
| | $n^a$ | All[a] | DCIS | T1a+b (≤ 10 mm) | T1c (11–20 mm) | T2+ (> 20 mm) | Tx NC |
| Underlying incidence | 2809 | 100 | 3.3 | 7.1 | 31.6 | 55.1 | 2.9 |
| Initial detection 1990–1993 | 3639 | 100 | 13.8 | 26.3 | 37.0 | 19.5 | 3.4 |
| Subsequent detection 1990–1993 | 712 | 100 | 14.6 | 29.2 | 35.4 | 18.8 | 2.0 |
| Interval cancers 1st year[b] | 464 | 100 | 2.0 | 11.5 | 41.3 | 39.2 | 5.8 |
| Interval cancers 2nd year[b] | 915 | 100 | 2.0 | 11.3 | 33.2 | 45.9 | 7.5 |
| Breast cancers 1990–1993 not (yet) screened women | 11 895 | 100 | 4.3 | 8.2 | 32.4 | 48.9 | 6.2 |

[a]Including Tx tumours and not classified breast cancers (NC). [b]Including both interval cancers after initial screens and interval cancers after subsequent screens because these data were not separately available.

In not-screened women, about half of all diagnosed cancers were more than 20 mm in size (T2+). Compared with 1989, breast cancer diagnosed in not-screened women during 1990–1993 seemed to have shifted towards a more favourable tumour size distribution, but the higher proportion of Tx-tumours and not-classified cancers (6.2% vs 2.9%) should be borne in mind. In screen-detected cancers, more than 75% were DCIS or invasive cancer not larger than 20 mm in size. The tumour size distribution was slightly more favourable in subsequent screens. In interval cancers, the proportion of large invasive cancers was about twice as high as in screen-detected cancers but still lower than in not-screened women, especially in the first year after screening. In the second year after screening the tumour size distribution worsened and started to look more like that of breast cancers in not-screened women.

### Comparison with UK programme

Figure 1 compares relative incidences of invasive screen-detected cancers and proportionate incidences of invasive interval cancers in The Netherlands and the UK regions North West and East Anglia for initial screens in women aged 50–64 years. The Dutch programme detected relatively less invasive cancers than the two UK regions. In the first year after screening, the proportionate incidence of invasive interval cancers was similar. In the second year after screening this incidence was clearly lower in the North West region (48% vs 55%).

Figure 2 presents the per cent tumour size distribution, including DCIS, for The Netherlands and the North West region. In the North West, 55% of all detected cancers were in situ carcinomas or small invasive cancers ≤ 10 mm against 42% in The Netherlands. Whereas first year interval cancers in The Netherlands showed a slightly more favourable tumour size distribution, in the second year after screening the reverse was the case, and more than half of the interval cancers were large (T2+).

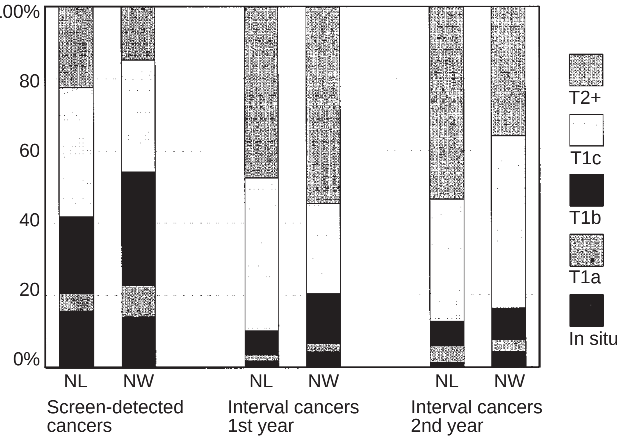

**Figure 2** Tumour size distribution (including in situ carcinomas) of screen-detected cancers and interval cancers in the first year and in the second year after initial screening, the Netherlands (NL) and the North West region (NW), women aged 50–64 years

### DISCUSSION

This is the first study with detailed information on interval cancers from a nationwide breast cancer screening programme. Major efforts were required of the regional screening organizations and cancer registries to carry out the linkage procedure and to estimate the follow-up time of all individual women. This enabled us to calculate interval cancer rates per woman-years, which is a better approach than expressing them per number of screened women (Prior, 1996). Otherwise, not only loss to follow-up due to deaths and relocations would not be taken into account but also the fact that in the Dutch programme the average screening interval was one month shorter than 2 years (Fracheboud et al, 1998).

In the first interval after the initial screen, 1002 interval cancers (invasive and in-situ), or 0.95 per 1000 woman-years, were diagnosed. This was less than expected (1.00 per 1000) but we cannot exclude a slight under-reporting of interval cancers due to overlap between the working areas of screening organization and regional cancer register. An indication for this may be the higher rates observed in Limburg, one of the Dutch regions where screening area and regional cancer register completely coincide (Schouten et al, 1998).

The tumour size distribution of interval cancers was clearly less favourable than that of screen-detected cancers but more favourable than in not-screened women, particularly in the first year after screening. In the second year the proportion of large invasive tumours > 20 mm increased and the tumour size distribution started to look more like that in not-screened women. The figures concerning T2+ tumours in Table 2 and Table 3 show an annual reduction in the number of advanced cancers of 16% during the 2-year prevalence screen period and of 44% during an incidence screen cycle. However, the higher proportion of cancers of unknown tumour size in interval cancers calls for a careful interpretation, depending on which assumption about the tumour size distribution of cancers of unknown tumour size is made. If most of these cancers were advanced tumours, the reduction in advanced tumours would be less during the described period. Nevertheless, the observed reduction in the number of advanced cancers is likely to be an important early indicator of the expected mortality reduction that is illustrated by the 5% lower breast cancer mortality in The Netherlands since the start of the programme (van den Akker-van Marle, 1999).

Because of differences in targeted age groups and/or the lack of incidence figures, comparison with other screening programmes had to be limited to regional outcomes of the UK screening programme. Despite a relatively higher underlying incidence, the Dutch overall detection rate of 5.73 per 1000 (50–64 years) was lower than those reported by different UK regions of 5.9–6.7 per 1000 (Woodman et al, 1995; Garvican and Littlejohns, 1996; Sylvester et al, 1997) or in the nationwide UK programme (Moss et al, 1995). Compared to the North West region (Figure 2), the Dutch programme not only detected fewer breast cancers but also relatively fewer small invasive cancers. This is somewhat surprising as in the past, two-view mammography and double reading have not been used routinely in the UK. Moreover, in the UK programme microinvasive cancer are usually excluded from invasive cancers, whereas in The Netherlands they are classified as small invasive cancer (T1a). With regard to interval cancers, there were less differences in relative (proportionate) incidences between the Dutch nationwide programme and the outcomes of two UK regions. All of them showed substantially higher proportionate incidences than the Swedish Two County Study (Tabár et al, 1987).

However, the comparisons of relative (proportionate) incidence should be interpreted with caution. In the Dutch setting breast cancer in non-attending and non-invited women cannot be identified separately due to legal limitations. For this reason, the underlying incidence is defined as the known incidence before the start of the screening programme. However, this incidence is based on historical data of only 1 year (1989, the first year of national coverage of the National Cancer Registry), and, because of the exclusion of the two regions where the pilot programmes took place, not on complete nationwide data. It is therefore difficult to estimate how the incidence would have developed without a screening programme (Prior et al, 1996). Even without organized screening, regional cancer registry data showed an increasing breast cancer incidence in the late 1980s (Nab et al, 1993). The slightly higher incidence rate in not (yet) screened women in 1990–1993 compared with 1989 suggests a steady increase of the underlying incidence. This may be caused by an increasing breast awareness and a general trend to earlier detection of breast cancer given the 37% increase of in situ cancers and the 22% increase of small invasive tumours of ≤10 mm (Table 2).

Nevertheless, efforts to improve the detection of small invasive cancers should be considered, especially at subsequent screens because the observed interval cancer rates in subsequent screens were slightly higher than expected and may be still higher supposing some underreporting of interval cancers. The Dutch programme, however, has as a low-referral rate, with only 1% referrals for further assessment of all women screened during 1990–1996 (NETB, 1997). In the UK programme with reasonably comparable interval cancer rates, the referral rate was 6%. The Dutch pilot projects had already tried to find an optimal balance between too many women unnecessary referred and too few early cancers detected. The national screening programme adopted this emphasis on low false-positive rates. Nonetheless, the present findings suggest that the emphasis should be more on preventing false-negatives, perhaps at the price of higher referral rates.

### ACKNOWLEDGEMENTS

The national evaluation of breast cancer screening is funded by the Health Insurance Executive Board (Ziekenfondsraad), Amstelveen. The authors thank the regional screening organizations and comprehensive cancer centres for providing the data: Stichting Kankerpreventie IKA/Integraal Kankercentrum Amsterdam; Stichting Kankerpreventie en -screening Limburg/Integraal Kankercentrum Limburg, Maastricht; Stichting Preventicon voor de Vroege Opsporing van Borstkanker Midden-Nederland/ Integraal Kankercentrum Midden-Nederland, Utrecht; Stichting Bevolkingsonderzoek Borstkanker Noord-Nederland/Integraal Kankercentrum Noord, Groningen; Stichting Vroege Opsporing Kanker Oost-Nederland/Integraal Kankercentrum Oost, Nijmegen; Stichting Vroege Opsporing Borstkanker/Integraal Kankercentrum Stedendriehoek Twente, Enschede; Stichting Bevolkingsonderzoek Borstkanker Zuid-West-Nederland/ Integraal Kankercentrum Rotterdam, Rotterdam; Stichting Kankerpreventie West-Nederland/Integraal Kankercentrum West, Leiden; Stichting Bevolkingsonderzoek Borstkanker Zuid, 's-Hertogenbosch/Integraal Kankercentrum Zuid, Eindhoven.

### REFERENCES

Akker-van Marle ME van den, Koning HJ de, Boer R and Maas PJ van der (1999) Reduction in breast cancer mortality due to the introduction of mass screening in the Netherlands; comparison with the UK. *J Med Screening* **6**: 30–34

Boer R, Koning HJ de, Threlfall A, Warmerdam P, Street A, Friedman E and Woodman C (1998) Cost effectiveness of shortening screening interval or extending age range of NHS breast screening programme: computer simulation study. *Br Med J* **317**: 376–379

Brekelmans CTM, Collette HJA, Collette C, Fracheboud J and Waard F de (1992) Breast cancer after a negative screen: follow-up of women participating in the DOM screening programme. *Eur J Cancer* **28A**: 893–895

Day N, McCann J, Camilleri-Ferrante C, Britton P, Hurst G, Cush S, Duffy S on behalf of the Quality Assurance Management Group of the East Anglian Breast Screening Programme (1995) Monitoring interval cancers in breast screening programmes: the East Anglian experience. *J Med Screening* **2**: 180–185

Faux AM, Richardson DC, Lawrence GM, Wheaton ME and Wallis MG (1997) Interval breast cancers in the NHS Breast Screening Programme: does the current definition exclude too many? *J Med Screening* **4**: 169–173

Fracheboud J, Koning HJ de, Beemsterboer PMM, Boer R, Hendriks JHCL, Verbeek ALM, Ineveld BM van, Bruyn AE de and Maas PJ van der (1998) Nation-wide breast cancer screening in the Netherlands: results of initial and subsequent screening 1990–1995. *Int J Cancer* **75**: 694–698

Garvican L and Littlejohns P (1996) An evaluation of the prevalent round of the breast screening programme in South East Thames, 1988–1993: achievement of quality standards and population impact. *J Med Screening* **3**: 123–128

Klemi PJ, Toikkanen S, Räsänen O, Parvinen I and Joensuu H (1997) Mammography screening interval and the frequency of interval cancers in a population-based screening. *Br J Cancer* **75(5)**: 762–766

Koning HJ de, Ineveld BM van, Oortmarssen GJ van, Haes JCJM de, Collette HJA, Hendriks JHCL and Maas PJ van der (1991) Breast cancer screening and cost-effectiveness; policy alternatives, quality of life considerations and the possible impact of uncertain factors. *Int J Cancer* **49**: 531–537

Koning HJ de, Fracheboud J, Boer R, Verbeek ALM, Collette HJA, Hendriks JHCL, Ineveld BM van, Bruyn AE and PJ van der Maas (National Evaluation Team for Breast cancer screening) (1995*a*). Nation-wide breast cancer screening in

© 1999 Cancer Research Campaign the Netherlands: support for breast-cancer mortality reduction. *Int J Cancer* **60**: 777–780

Koning HJ de, Boer R, Warmerdam PG, Beemsterboer PMM and Maas PJ van der (1995*b*) Quantitative interpretation of age-specific mortality reductions from the Swedish breast cancer screening trial. *J Natl Cancer Inst* **87**: 1217–1223

Moss SM, Coleman DA, Ellman R, Chamberlain J, Forrest APM, Kirkpatrick AE, Thomas BA and Price JL (1993) Interval cancers and sensitivity in the screening centres of the UK trial of early detection of breast cancer. *Eur J Cancer* **29A**: 225–258

Moss SM, Michel M, Patnick J, Johns L, Blanks R and Chamberlain J (1995) Results from the NHS breast screening programme 1990–1993. *J Med Screening* **2**: 186–190

Nab HW, Voogd AC, Crommelin MA, Kluck HM, Heijden LH van der and Coebergh JWW (1993). Breast cancer in south-east Netherlands, 1960–1989: trends in incidence and mortality. *Eur J Cancer* **29A**: 1557–1560

NETB – National Evaluation Team for Breast cancer screening (1995) National evaluation of breast cancer screening in the Netherlands (IV). Rotterdam: Department of Public Health, Erasmus University Rotterdam (in Dutch)

NETB – National Evaluation Team for Breast cancer screening (1997) National evaluation of breast cancer screening in the Netherland (VI). Rotterdam: Department of Public Health, Erasmus University Rotterdam (in Dutch, with summary in English)

NCR – Netherlands Cancer Registry (1992) Incidence of cancer in the Netherlands 1989 SIG Health Care Information, Utrecht

Oortmarssen GJ van, Habbema JDF, Maas PJ van der, Koning HJ de, Collette HJA, Verbeek ALM, Geerts AT and Lubbe KTN (1990) A model for breast cancer screening. *Cancer* **66**: 1601–1612

Peeters PHM, Verbeek ALM, Hendriks JHCL, Holland R, Mravunac M and Vooijs GP (1989) The occurrence of interval cancers in the Nijmegen screening programme. *Br J Cancer* **59**: 929–932

Prior P, Woodman CBJ, Wilson S and Threlfall AG (1996) Reliability of underlying incidence rates for estimating the effect and efficiency of screening for breast cancer. *J Med Screening* **3**: 119–122

Schouten LJ, Rijke JM de, Schlangen JT and Verbeek ALM (1998) Evaluation of the effect of breast cancer screening by record linkage with the cancer registry, the Netherlands. *J Med Screening* **5**: 37–41

Sylvester PA, Davies JD, Vipond MN, Webb AJ, Kutt E and Farndon JR (1997) A comparative audit of prevalent, incident and interval cancers in the Avon breast screening programme. *Ann R Coll Surg Engl* **79**: 272–275

Tabár L, Faberberg G, Day NE and Holmberg L (1987) What is the optimum interval between mammographic screening examinations? An analysis based on the latest results of the Swedish two-county breast cancer screening trial. *Br J Cancer* **55**: 547–551

Vitak B, Stål O, Månson JC, Thomas BA, Arnesson LG, Ekelund L, Måre K, Nordenskjöld B, Källström AC and Bång H (1997) Interval cancers and cancers in non-attenders in the Östergötland mammographic screening programme. Duration between screening and diagnosis, S-phase fraction and distant recurrence. *Eur J Cancer* **33**: 1453–1460

Woodman CBJ, Threlfall AG, Boggis CRM and Prior P (1995) Is the three-year breast screening interval too long? Occurrence of interval cancers in NHS breast screening programme's north western region. *Br Med J* **310**: 224–226

© 1999 Cancer Research Campaign

## References

[PDF_00481] Boer R., de Koning H., Threlfall A., Warmerdam P., Street A., Friedman E., Woodman C. (1998). Cost effectiveness of shortening screening interval or extending age range of NHS breast screening programme: computer simulation study.. BMJ.

[PDF_00484] Brekelmans C. T., Collette H. J., Collette C., Fracheboud J., de Waard F. (1992). Breast cancer after a negative screen: follow-up of women participating in the DOM Screening Programme.. Eur J Cancer.

[PDF_00487] Day N., McCann J., Camilleri-Ferrante C., Britton P., Hurst G., Cush S., Duffy S. (1995). Monitoring interval cancers in breast screening programmes: the east Anglian experience. Quality Assurance Management Group of the East Anglian Breast Screening Programme.. J Med Screen.

[PDF_00491] Faux A. M., Richardson D. C., Lawrence G. M., Wheaton M. E., Wallis M. G. (1997). Interval breast cancers in the NHS Breast Screening Programme: does the current definition exclude too many?. J Med Screen.

[PDF_00494] Fracheboud J., de Koning H. J., Beemsterboer P. M., Boer R., Hendriks J. H., Verbeek A. L., van Ineveld B. M., de Bruyn A. E., van der Maas P. J. (1998). Nation-wide breast cancer screening in The Netherlands: results of initial and subsequent screening 1990-1995. National Evaluation Team for Breast Cancer Screening.. Int J Cancer.

[PDF_00498] Garvican L., Littlejohns P. (1996). An evaluation of the prevalent round of the breast screening programme in south east Thames, 1988-1993: achievement of quality standards and population impact.. J Med Screen.

[PDF_00501] Klemi P. J., Toikkanen S., Räsänen O., Parvinen I., Joensuu H. (1997). Mammography screening interval and the frequency of interval cancers in a population-based screening.. Br J Cancer.

[PDF_00520] Moss S. M., Coleman D. A., Ellman R., Chamberlain J., Forrest A. P., Kirkpatrick A. E., Thomas B. A., Price J. L. (1993). Interval cancers and sensitivity in the screening centres of the UK trial of early detection of breast cancer.. Eur J Cancer.

[PDF_00524] Moss S. M., Michel M., Patnick J., Johns L., Blanks R., Chamberlain J. (1995). Results from the NHS breast screening programme 1990-1993.. J Med Screen.

[PDF_00527] Nab H. W., Voogd A. C., Crommelin M. A., Kluck H. M., vd Heijden L. H., Coebergh J. W. (1993). Breast cancer in the southeastern Netherlands, 1960-1989: trends in incidence and mortality.. Eur J Cancer.

[PDF_00542] Peeters P. H., Verbeek A. L., Hendriks J. H., Holland R., Mravunac M., Vooijs G. P. (1989). The occurrence of interval cancers in the Nijmegen screening programme.. Br J Cancer.

[PDF_00545] Prior P., Woodman C. B., Wilson S., Threlfall A. G. (1996). Reliability of underlying incidence rates for estimating the effect and efficiency of screening for breast cancer.. J Med Screen.

[PDF_00548] Schouten L. J., de Rijke J. M., Schlangen J. T., Verbeek A. L. (1998). Evaluation of the effect of breast cancer screening by record linkage with the cancer registry, The Netherlands.. J Med Screen.

[PDF_00551] Sylvester P. A., Vipond M. N., Kutt E., Davies J. D., Webb A. J., Farndon J. R. (1997). A comparative audit of prevalent, incident and interval cancers in the Avon breast screening programme.. Ann R Coll Surg Engl.

[PDF_00554] Tabár L., Faberberg G., Day N. E., Holmberg L. (1987). What is the optimum interval between mammographic screening examinations? An analysis based on the latest results of the Swedish two-county breast cancer screening trial.. Br J Cancer.

[PDF_00558] Vitak B., Stål O., Månson J. C., Thomas B. A., Arnesson L. G., Ekelund L., Måre K., Nordenskjöld B., Källström A. C., Bång H. (1997). Interval cancers and cancers in non-attenders in the Ostergötland Mammographic Screening Programme. Duration between screening and diagnosis, S-phase fraction and distant recurrence.. Eur J Cancer.

[PDF_00563] Woodman C. B., Threlfall A. G., Boggis C. R., Prior P. (1995). Is the three year breast screening interval too long? Occurrence of interval cancers in NHS breast screening programme's north western region.. BMJ.

[PDF_00517] de Koning H. J., Boer R., Warmerdam P. G., Beemsterboer P. M., van der Maas P. J. (1995). Quantitative interpretation of age-specific mortality reductions from the Swedish breast cancer-screening trials.. J Natl Cancer Inst.

[PDF_00504] de Koning H. J., van Ineveld B. M., van Oortmarssen G. J., de Haes J. C., Collette H. J., Hendriks J. H., van der Maas P. J. (1991). Breast cancer screening and cost-effectiveness; policy alternatives, quality of life considerations and the possible impact of uncertain factors.. Int J Cancer.

[PDF_00540] van Oortmarssen G. J., Habbema J. D., van der Maas P. J., de Koning H. J., Collette H. J., Verbeek A. L., Geerts A. T., Lubbe K. T. (1990). A model for breast cancer screening.. Cancer.

[PDF_00477] van den Akker-van Marle E., de Koning H., Boer R., van der Maas P. (1999). Reduction in breast cancer mortality due to the introduction of mass screening in The Netherlands: comparison with the United Kingdom.. J Med Screen.

